# Impact of Disclosing to Patients the Use of Antiretroviral Resistance Testing Results for Molecular HIV Surveillance: A Randomized Experiment in 2 National Surveys

**DOI:** 10.2196/64663

**Published:** 2025-04-11

**Authors:** Jeremy Sugarman, Juli Bollinger, Jose Agostini, Kevin Weinfurt, Gail Geller, Sheethal Jose, Marissa Hannah, O. Winslow Edwards, Leslie Meltzer Henry, Travis Sanchez

**Affiliations:** 1 Berman Institute of Bioethics, Johns Hopkins University, 1809 Ashland Avenue, Baltimore, MD, 21205, United States, 1 410-614-5634; 2 School of Medicine, Johns Hopkins University, Baltimore, MD, United States; 3 Department of Epidemiology, Rollins School of Public Health, Emory University, Atlanta, GA, United States; 4 Department of Population Health Sciences, Duke University School of Medicine, Durham, NC, United States; 5 Department of Health Policy and Management, Johns Hopkins Bloomberg School of Public Health, Baltimore, MD, United States; 6 Carey School of Law, University of Maryland, Baltimore, MD, United States

**Keywords:** HIV, disclosure, consent, antiretroviral, HIV gene, ARVRT, molecular HIV surveillance, antiretroviral resistance, antiretroviral resistance testing, molecular HIV, HIV surveillance, public health, people living with HIV, web-based survey, United States, AMIS, TWIST, prevention and care, American Men’s Internet Survey, Transgender Women’s Internet Survey and Testing

## Abstract

**Background:**

Molecular HIV surveillance (MHS) can be used to help identify and respond to emerging clusters of rapidly spreading HIV transmissions, a practice known as cluster detection and response (CDR). In the United States, MHS relies on HIV gene sequences obtained from routine clinical antiretroviral resistance testing (ARVRT). By law, ARVRT results are reported to public health agencies for MHS and individuals are not asked for their specific consent to do so. This practice has raised ethical concerns, including the lack of consent for, and transparency surrounding, public health uses of these clinical data. Such concerns have spurred debate and could have a chilling effect on the willingness of people living with HIV to agree to ARVRT when recommended clinically and jeopardize the utility of MHS-informed HIV prevention efforts. In response to the lack of routine disclosure of use of ARVRT results for MHS, in 2022, the Presidential Advisory Council on HIV/AIDS (PACHA) issued a resolution calling on the US Centers for Disease Control to “require that providers explain MHS/CDR and the laboratory test results that are collected and used in these surveillance activities to their patients.”

**Objective:**

This study aimed to examine the effect of clinician disclosure of the public health uses of ARVRT results for MHS versus clinician nondisclosure on patient willingness to undergo recommended ARVRT.

**Methods:**

We conducted a randomized survey experiment examining the effect of clinician disclosure of the public health uses of ARVRT results for MHS versus clinician nondisclosure (the current standard of care) and subsequent discovery of such uses through a “trusted media source” on patient willingness to undergo recommended ARVRT. Study participants were respondents to 1 of 2 national web-based surveys conducted annually in the United States: the American Men’s Internet Survey (AMIS) and the Transgender Women’s Internet Survey and Testing (TWIST).

**Results:**

Overall, 4348 AMIS participants (n=2151 disclosure; n=2197 nondisclosure) and 3314 TWIST participants (n=1670 disclosure; n=1644 nondisclosure) completed survey items regarding the randomly assigned vignettes. The majority were willing to undergo ARVRT regardless of which vignette they saw (1670/2151, 82.7% [AMIS] and 1326/1670, 80.8% [TWIST] in the disclosure group; and 1399/2197, 68% [AMIS] and 1101/1674, 68.45% [TWIST] in the nondisclosure group) after later learning about public health uses of ARVRT results.

**Conclusions:**

The majority of respondents expressed willingness to undergo ARVRT even with disclosure of public health uses of these data, but willingness markedly decreased when learning about these uses after the fact, highlighting the importance of transparency in MHS programs. Accordingly, in line with the ethical principle of respect for autonomy and the likelihood that the potential public health benefits of MHS programs will not be compromised, consideration should be given to encouraging clinicians to disclose public health uses of ARVRT at the time ARVRT is recommended.

## Introduction

Molecular HIV surveillance (MHS) is used in public health to help identify and respond to clusters of rapidly spreading HIV transmissions, a practice known as cluster detection and response (CDR). In the United States, MHS relies on partial HIV gene sequences obtained from antiretroviral resistance testing (ARVRT) performed during routine clinical care of people living with HIV [1]. As opposed to time-space clusters that rely on the identification of an increase in new HIV diagnoses in a geographic area, MHS/CDR uses ARVRT data to identify emerging clusters of highly similar HIV sequences, which is an indicator of rapid HIV transmission [2]. Research has demonstrated that rapidly growing clusters contribute disproportionally to new HIV infections [3]. The use of MHS/CDR allows health departments to direct and prioritize resources toward groups experiencing rapid HIV transmission [2].

In the interests of public health and as is the case with other reportable conditions, ARVRT results are reported to public health agencies for MHS and individuals are not asked for their specific consent to do so. In 2018, the US Centers for Disease Control and Prevention (CDC) mandated that health departments include MHS in their CDR procedures; and in 2019, the use of MHS for CDR was identified as a pillar of the US Ending the HIV Epidemic initiative [4].

While difficult to quantify, the CDC has cited beneficial results of using MHS for CDR, including reduction in new HIV diagnoses, improved HIV testing and diagnoses, increased pre-exposure prophylaxis uptake, expanded syringe services program use, and improved linkage to care [5-7]. Nevertheless, current practices regarding such uses have raised ethical and social concerns regarding disclosure, consent, privacy, stigma, and the potential for HIV-related criminal prosecution [8-11]. Critics have argued that the identification of individuals in transmission clusters for intervention and prevention services, and the publicity surrounding those networks, could potentially further increase stigma and legal risk for marginalized individuals and groups including people of color, sex workers, and those already subject to enhanced policing and surveillance [12,13]. Several advocacy organizations have opposed using MHS for CDR [12,13], with some calling upon the CDC to halt its use altogether [14], citing concerns that the personal and medical information of people living with HIV are being used for surveillance purposes without meaningful engagement of people living with HIV in program planning, individuals’ knowledge about how their ARVRT results are used in surveillance activities, and lack of informed consent. In response to these concerns, the Presidential Advisory Council on HIV/AIDS (PACHA) passed a nonbinding resolution in 2022 calling for substantial action by public health agencies and others, including the need to meaningfully engage with communities regarding MHS to minimize harms associated with its implementation, to gather evidence concerning its use, to obtain consent for it, to review criminal and public health laws that disparately treat people living with HIV, and to address punitive laws related to HIV criminalization [15]. To deal with the lack of transparency surrounding the use of clinical ARVRT results for MHS/CDR, the resolution called upon CDC to “require that providers explain MHS/CDR and the laboratory test results that are collected and used in these surveillance activities to their patients” [15].

While use of MHS continues to spur vigorous debate, to date there has not been a systematic assessment of how the risks and benefits of MHS are perceived among people living with HIV and people without HIV at increased risk of acquiring it. If left unidentified and unaddressed, these perceptions and risks could conceivably have a chilling effect on the willingness of people living without HIV at increased risk to pursue HIV testing and people living with HIV to agree to ARVRT when recommended clinically as well as decrease trust in both the health care system and public health.

To this end, our multidisciplinary team is conducting a mixed-methods, multiphase research project titled, “Study of Ethics and Stakeholder Attitudes towards Molecular Epidemiology (SESAME)” to collect data from a wide range of stakeholders to inform developing policies and practices.

In this report, we describe the results of a randomized survey experiment examining the effect of clinician disclosure of the public health uses of ARVRT results for MHS versus clinician nondisclosure and subsequent discovery of such uses through a “trusted media source” on patient willingness to undergo recommended ARVRT.

## Methods

### Study Population and Procedures

Participants in this study were respondents to 1 of 2 national web-based surveys fielded annually in the United States: The American Men’s Internet Survey (AMIS) conducted from October 2022 to October 2023 and the Transgender Women’s Internet Survey and Testing (TWIST) conducted from June 2022 to October 2023. AMIS is a cross-sectional study that seeks participants from eligible cisgender gay, bisexual, same gender loving, and other men who have sex with men (GBMSM) to provide information useful for monitoring trends in sexual health [16]. Briefly, participants were recruited through online advertisements on social media sites and apps among the target population of GBMSM. All participants who clicked on advertisements links were taken to the survey website for screening. Respondents were eligible for AMIS if they were at least 15 years of age, reported male sex at birth and male gender identity, resided in the United States, and reported ever having oral or anal sex with a male partner. TWIST involves transgender women and transfeminine nonbinary people assigned male at birth [17]. Recruitment for TWIST was similar to AMIS and was conducted with advertisements on social media platforms and apps popular among the target population. Respondents were eligible for TWIST if they were at least 15 years of age, reported male sex at birth and feminine (woman or transgender woman) gender, resided in the United States, and reported ever having oral, anal, or vaginal sex.

**Figure 1.**
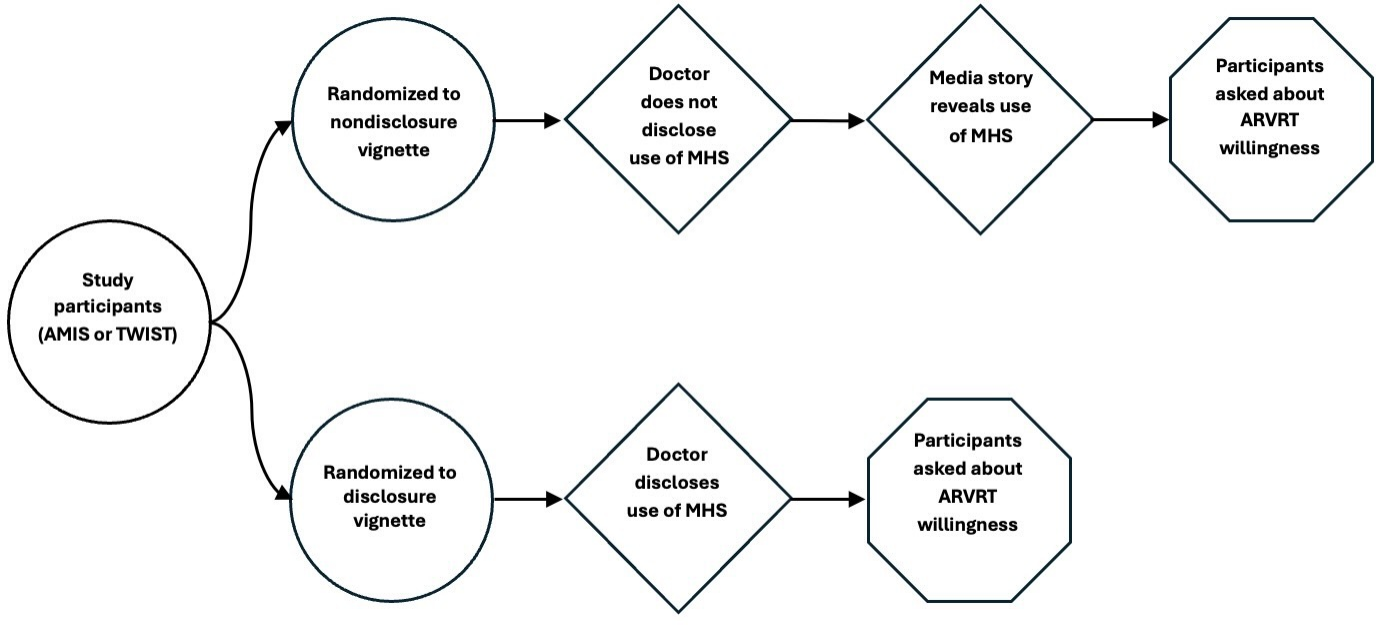
Visual depiction of the survey experiment. Respondents were randomly assigned to view 1 of 2 hypothetical vignettes that both set the scenario as them being someone living with HIV seeing a clinician who asked them about their willingness to undergo standard antiretroviral resistance testing (ARVRT). Survey arms differed by whether the treating clinician disclosed public health use of ARVRT results for molecular HIV surveillance or the respondent learns about this later through a media story. AMIS: American Men’s Internet Survey; MHS: molecular HIV surveillance; TWIST: Transgender Women’s Internet Survey and Testing.

In each study, participants were randomly assigned to 1 of 2 hypothetical vignettes that both set the scenario as them being someone living with HIV seeing a clinician who asked them about their willingness to undergo standard ARVRT. This scenario was the same regardless of the participant’s self-reported HIV status in the survey (Figure 1.).

In the “disclosure” vignette, the clinician initially discloses the public health reporting and MHS associated with ARVRT, and then participants are asked about their willingness to have the test performed. In the “nondisclosure” vignette, which generally reflects the current standard of care, the public health reporting and MHS practices are not initially shared with the participant, and they are asked about their willingness to undergo ARVRT. Then, they are told about a “media story” that indicates ARVRT results are reported to public health agencies for MHS. Willingness to have ARVRT was then asked again. (The text of the 2 vignettes and questions regarding them are available in the MultimediaAppendices 1  2)

Before fielding, the vignette descriptions and survey questions were iteratively tested and revised through a series of 12 cognitive interviews with MSM to test for clarity and comprehension. Potential interviewees were recruited using a convenience sample of previous AMIS respondents who provided their email addresses so they could be contacted regarding participation in future studies. Cognitive interviews were conducted virtually over Zoom (Zoom Communications) by trained interviewers using a cognitive interview guide (Multimedia Appendix 3) developed by the research team.

### Measures

The dependent measure in this study is willingness to undergo ARVRT. For the disclosure vignette, this was asked only once. For the nondisclosure vignette, the measure of interest for this study was the willingness to undergo ARVRT both before and after the participant learned of the public health uses through the media story. The response scale was “very unwilling, unwilling, neither willing nor unwilling, willing, and very willing.” For this analysis, responses were dichotomized to combine “very willing” and “willing” responses into “willing” and all other responses as “unwilling.”

The primary independent measure was the randomization to either the disclosure or nondisclosure vignette. Other independent measures were participants’ race and ethnicity, self-reported HIV status, and anticipated health care stigma. Race was captured with a question asking participants to select all races that apply from a list of possible options and a separate yes/no question about Hispanic or Latinx ethnicity. For this analysis, responses were categorized into Hispanic or Latinx, non-Hispanic Black, non-Hispanic White, and other or multiple race [16]. HIV status was self-reported based on the result of respondents’ most recent HIV test. Anticipated health care stigma is a variable created with responses to a pair of questions about whether they ever “avoided” or “felt afraid” getting health care services because of how they would be treated [18]. AMIS participants were asked about stigma related to having sex with men. TWIST participants were asked about anticipated stigma related to who they have sex with or their gender identity. Participants who answered yes to either of these questions were recorded as having anticipated health care stigma, and those who reported no fear or avoidance of health care services were labeled as not having anticipated stigma.

### Analyses

Analyses were conducted with unduplicated successful survey responses from participants who answered the ARVRT willingness questions. The procedures for deduplication and successful completion metrics have been previously described [16,17]. Comparisons in dichotomous willingness to have ARVRT by vignette were calculated as prevalence ratios with 95% CI. Multivariable modeling was not deemed necessary for this randomized study with no failure of randomization detected in participant characteristics between arms. Prevalence ratios greater than the null (1) would indicate that those in the disclosure vignette were more willing to have ARVRT than those in the nondisclosure vignette. To explore heterogeneity in the effects, these comparisons were also stratified by participant race and ethnicity, self-reported HIV status, and anticipated health care stigma. Analyses were conducted in SAS version 9.4 (SAS Institute) and significance level was α=.05.

### Ethical Considerations

#### Cognitive Interviews

Cognitive interview participants were sent the oral consent script by email in advance of the virtual interview and provided oral consent before beginning the interview. Each participant was provided US $20 for completing the virtual interview. All data related to the cognitive interviews were stored in a secure analytic environment at Johns Hopkins University in a project folder accessible only to study team members who conducted the interviews using password-protected computers. The cognitive interview protocol was reviewed and approved as an expedited study by the Johns Hopkins Medicine Institutional Review Board (IRB00331966).

#### American Men’s Internet Survey and Transgender Women’s Internet Survey

AMIS and TWIST participants provided online informed consent before beginning the online self-administered survey. AMIS participants were not compensated; TWIST participants were compensated US $10 for survey completion. The AMIS and TWIST surveys are administered on a secure online survey platform (Alchemer) and include questions on demographic characteristics, sexual and substance use behaviors, HIV testing history, stigma and discrimination, and mental health. Personal identifiers other than email address and IP address are not collected. Original study data are stored on secure network drives with access restricted to authorized study staff. Datasets for analyses are deidentified, and no identifiable information is presented in this manuscript or supplemental files. The AMIS and TWIST studies were reviewed and approved by Emory University’s human subjects research review panel (protocol 47676 and 108784, respectively).

All project data are protected by a US federal Certificate of Confidentiality that prevents court-ordered disclosure of research data.

## Results

### American Men’s Internet Survey

Overall, 4348 AMIS participants completed the vignette items (n=2151 disclosure vignette and n=2197 nondisclosure vignette) (Table 1). Most were aged 30 years or older and non-Hispanic White. People living with HIV comprised 12.2% (529/4348) of AMIS participants. Among those who answered the anticipated health care stigma question (n=2260), 33.9% (767/2260) reported ever experiencing this stigma. The majority were willing to undergo ARVRT regardless of which vignette they saw (1670/2151, 82.7% in the disclosure group; and 2186/2197, 92.4% in the nondisclosure group before the media story and 1399/2197, 68% after the media story), but those in the disclosure group were significantly more likely to be willing to have ARVRT compared with the nondisclosure group after the media story disclosed MHS uses of ARVRT information (prevalence ratio=1.22, 95% CI 1.17-1.26). In stratifications by age, race and ethnicity, HIV status, and anticipated health care stigma, the effects were all in the same direction and of similar magnitude to the overall finding.

**Table 1.** Willingness to have HIV antiretroviral resistance testing under randomized scenarios of clinician disclosure of HIV molecular epidemiology by key participant characteristics, United States Cisgender Men Who Have Sex with Men and Transfeminine Persons, 2022‐2023.

Characteristic	Overall, n	Randomization group				Prevalence ratio (95% CI)
		Disclosure, n/N (%)		Nondisclosure^a^ (ref), n/N (%)		
All men who have sex with men (AMIS^b^ participants)	4348	1670/2151 (82.7)		1399/2197 (68)		1.22 (1.17-1.26)
Age (years)						
15‐24	335	121/169 (77.6)		106/166 (69.3)		1.12 (0.98-1.28)
25‐29	407	173/215 (85.2)		135/192 (74.6)		1.14 (1.03-1.27)
30‐39	1014	397/496 (85.4)		346/518 (70.8)		1.21 (1.13-1.29)
40+	2592	979/1271 (81.9)		812/1321 (65.8)		1.24 (1.19-1.31)
Race and ethnicity						
Non-Hispanic Black	473	161/221 (79.7)		163/252 (69.4)		1.15 (1.03-1.28)
Hispanic	441	162/222 (78.7)		143/219 (69.8)		1.13 (1.01-1.27)
Non-Hispanic White	3015	1195/1501 (84)		962/1514 (67.7)		1.24 (1.19-1.3)
Other	383	146/194 (80.2)		123/189 (70.3)		1.14 (1.01-1.29)
HIV status						
Positive	529	202/258 (82.1)		172/271 (67.5)		1.22 (1.1-1.35)
Negative or unknown	3819	1468/1893 (82.8)		1227/1926 (68.1)		1.22 (1.17-1.26)
Anticipated health care stigma						
Yes	767	277/374 (79.4)		234/393 (63.9)		1.24 (1.13-1.36)
No	1493	572/732 (83.9)		487/761 (68.7)		1.22 (1.15-1.3)
All transfeminine persons (TWIST^c^ participants)	3314	1326/1670 (80.8)		1101/1644 (68.4)		1.18 (1.13-1.23)
Age (years)						
15‐24	1126	475/568 (85.1)		375/558 (69.1)		1.22 (1.16-1.32)
25‐29	719	283/370 (77.5)		248/349 (71.9)		1.08 (0.99-1.18)
30‐39	900	343/442 (79)		314/458 (69.8)		1.13 (1.05-1.22)
40+	569	225/290 (79.2)		164/279 (60.3)		1.31 (1.17-1.47)
Race and ethnicity						
Non-Hispanic Black	280	97/140 (71.3)		71/140 (53.8)		1.33 (1.1-1.61)
Hispanic	299	116/150 (78.4)		101/149 (71.1)		1.1 (0.96-1.26)
Non-Hispanic White	2566	1052/1297 (82.5)		877/1269 (70.1)		1.18 (1.13-1.23)
Other	135	52/68 (77.6)		44/67 (63.1)		1.23 (0.98-1.54)
HIV status						
Positive	49	22/26 (88)		10/23 (45.5)		1.94 (1.2-3.13)
Negative or Unknown	3265	1304/1644 (80.7)		1091/1621 (68.7)		1.18 (1.13-1.22)
Anticipated health care stigma						
Yes	1111	435/552 (80.3)		363/559 (66)		1.22 (1.13-1.31)
No	531	207/257 (81.8)		201/274 (74.7)		1.1 (1-1.2)

a After media story.

b AMIS: American Men’s Internet Survey.

c TWIST: Transgender Women’s Internet Survey and Testing.

### Transgender Women’s Internet Survey

Overall, 3314 TWIST participants completed the vignette items (n=1670 disclosure vignette and n=1644 nondisclosure vignette). Most were aged 30 years or younger and non-Hispanic White (Table 1). People living with HIV comprised 1.5% (49/3314) of TWIST participants. Among those who answered the anticipated health care stigma question (n=1642), 68.4% (n=1111) reported ever experiencing this stigma. The majority were willing to undergo ARVRT regardless of which vignette they saw (1326/1670, 80.8% in disclosure and 1446/1674, 89.5% in the nondisclosure group before the media story and 1101/1674, 68.4% after the media story) but those in the disclosure group were significantly more likely to be willing to have ARVRT compared with the nondisclosure group after the media story disclosed MHS uses of ARVRT information (prevalence ratio=1.18; 95% CI=1.13-1.23). In stratifications by age and anticipated health care stigma, the changes in willingness were all in the same direction and of similar magnitude to the overall finding. The association between disclosure vignette and willingness to have ARVRT was even more pronounced for TWIST participants living with HIV (prevalence ratio=1.94; 95% CI=1.2-3.13) than those who reported being HIV negative or of unknown HIV status.

## Discussion

In this study with GBMSM and transgender women or transfeminine persons from across the United States, majorities of both study populations were willing to undergo ARVRT recommended by an HIV care clinician regardless of the requirement for public health reporting of those test results and the manner in which they learned about the reporting requirement. Despite these majorities, however, our results show that disclosing the public health reporting requirement to patients at the time ARVRT is ordered resulted in significantly higher willingness to pursue ARVRT than subsequently finding out about this reporting requirement from a “media story.” The disclosure-related disparities in willingness to pursue ARVRT were most evident among transfeminine persons who were Black or who were living with HIV infection.

Importantly, our data suggest that informing patients upfront that ARVRT results are shared with public health authorities has only a modest effect on ARVRT uptake. Higher levels of ARVRT uptake were reported among nondisclosure vignette respondents when asked before learning about the public health reporting requirement through the “media story” and, thus, when they were “unaware” of the reporting requirement. However, when those in the nondisclosure group later learned of the public health reporting requirement from a media story, their subsequent willingness to pursue testing dropped significantly, far below the level reported by those who were informed by the clinician at the time of testing (disclosure group). This steep drop in willingness to pursue recommended ARVRT among those in the nondisclosure group is not surprising and is likely attributed to respondents’ learning that their ARVRT results were shared with public health authorities without their knowledge.

Critics of MHS have advocated for incorporating informed consent into sharing ARVRT data with public health authorities for MHS, citing a lack of transparency about the process and concerns about individual privacy and autonomy. In their 2022 resolution PACHA urged “CDC to work with states and jurisdictions, alongside community members, providers, tribes, tribal epidemiology centers, members of networks of people living with HIV, and partner agencies within HHS to create a stronger system of informed consent around the uses of molecular HIV data” and called upon CDC to require states to “provide plain language notifications to individuals living with HIV on the types of surveillance and analyses being conducted and opt-out options for the use of MHS patient data for CDR activities” [15]. Nevertheless, while CDC’s recently updated HIV CDR Guidance for Health Departments includes calls for public health practitioners to be more transparent about the use of MHS, it does not endorse waivers or opting out of it [19].

In the absence of informed consent or an opt-out option for sharing ARVRT results with public health agencies for MHS, our data suggest the potential value of simply disclosing the public health use of ARVRT results to people living with HIV, preferably at the time testing is recommended. While disclosure is not equivalent to obtaining informed consent, upfront disclosure about public health reporting may resolve some of the ethical and community concerns regarding the lack for transparency about how personal data are used in MHS/CDR without jeopardizing the potential public health utility of MHS-informed HIV prevention efforts.

These findings also align with results from an earlier survey we conducted as part of our SESAME project where we examined 2 methods for explaining MHS to MSM living with HIV and those at-risk without HIV in the 2021 AMIS survey. Specifically, participants were randomly assigned to view a brief video or text explaining MHS. In the video group, respondents with high video engagement were less likely to be concerned about MHS; and in the text group, discomfort with MHS decreased as awareness of different public health activities increased [20].

The results of our study should be interpreted with some limitations in mind. First, while responses to hypothetical vignettes can be informative, it is unknown how well the findings translate to real-world situations. For example, we do not know why some participants in both arms of the study declined ARVRT testing at the time of their visit with the clinician. Clearly there were other factors at play besides the disclosure of public health use of these test results. Ideally, future demonstration projects in actual situations could assess the impact of disclosure on willingness to undergo ARVRT. In addition, unmeasured contextual factors may play a role in real-world decision making, but it is unlikely such factors would bias the results given randomization. Although the vignettes were simple, there may have been differential understanding of them based upon the vignette type. Of note, to overcome this possibility, we conducted extensive cognitive testing of the scenarios (as well as the response options) before fielding them.

Furthermore, AMIS and TWIST are online convenience sampling surveys and results are not generalizable to all people living with HIV and those without HIV at increased risk of acquiring it. The convenience sampling approach, even though conducted with multiple types of websites and social networking applications intended to increase sample diversity, increases the potential for selection biases and in ways that cannot be directly measured. Racial and ethnic minority persons in both AMIS and TWIST and younger persons in AMIS were also substantially underrepresented, potentially reducing our ability to discern key differences among these groups.

### Conclusions

The majority of respondents expressed willingness to undergo ARVRT even with disclosure of public health uses of these data, but willingness markedly decreased when people learn about these uses after the fact. Accordingly, in line with the ethical principle of respect to autonomy, and the likelihood that the potential public health benefits of MHS programs will not be compromised, consideration should be given to encouraging clinicians to disclose public health uses of ARVRT at the time testing is suggested.

## Supplementary material

10.2196/64663 Multimedia Appendix 1 American Men’s Internet Survey-Transgender Women’s Internet Survey and Testing vignette-disclosure.

10.2196/64663 Multimedia Appendix 2 American Men’s Internet Survey-Transgender Women’s Internet Survey and Testing vignette-no disclosure.

10.2196/64663 Multimedia Appendix 3 Cognitive interview guide for vignette survey.

## Abbreviations

AMIS: American Men’s Internet Survey
ARVRT: antiretroviral resistance testing
CDC: US Centers for Disease Control and Prevention
CDR: cluster detection and response
GBMSM: gay, bisexual, same gender loving, and other men who have sex with men
MHS: molecular HIV surveillance
PACHA: Presidential Advisory Council on HIV/AIDS
TWIST: Transgender Women’s Internet Survey and Testing
