# Supplementary material for: Impact of Disclosing to Patients the Use of Antiretroviral Resistance Testing Results for Molecular HIV Surveillance: A Randomized Experiment in 2 National Surveys
Source: JMIR Public Health Surveill. 2025 Apr 11;11:e64663. doi: 10.2196/64663 (PMC12007841; doi:10.2196/64663)
Supplement: Multimedia Appendix 2 [file publichealth-v11-e64663-s002.docx]

**2022 AMIS & TWIST Surveys – SESAME Vignette – NO DISCLOSURE**

Imagine you recently got tested for HIV and learned the test was **positive**. Now you are visiting a doctor at a local clinic to begin treatment.

The doctor suggests you start a once-a-day combination pill to treat HIV. The doctor tells you that this medicine works well for most people. However, for a small number of people, there are genetic changes in the HIV virus that make the medicine not work as well. To find out whether you are one of the people for whom the medicine might not work, the doctor orders an “HIV resistance test.” This blood test looks at the genetic makeup of the virus that you have. This test is done routinely as part of HIV treatment.

1. **We know that having a new diagnosis of HIV and thinking about treatment can be hard. For this question, we are specifically interested in your reaction to having the HIV resistance test done. How willing would you be to have the HIV resistance test done?**

- Very willing to have the resistance test done
- Somewhat willing to have the resistance test done
- Neither willing nor unwilling to have the resistance test done
- Somewhat unwilling to have the resistance test done
- Very unwilling to have the resistance test done

1. **How likely would you be to return to this doctor for continued care for HIV infection?**

- Very likely
- Somewhat likely
- Neither likely nor unlikely
- Somewhat unlikely
- Very unlikely

Now imagine that a while after your initial clinic visit with the doctor when you had the HIV resistance test done, you learned from a trusted media source that the results of HIV resistance tests are reported and sent automatically to public health agencies by the laboratory that did the test.

Patients are not asked to give consent for automatic reporting of HIV resistance test results because it is required by law, just like the reporting of all positive HIV test results. All of this information is used in a practice called “molecular surveillance” that allows public health agencies to understand how HIV is spreading in the community.

1. **What is your reaction to finding out that your HIV resistance test results would be sent to public health agencies?**

- I have no reaction.
- I am okay with this.
- I have a problem with this.

[If “I have a problem with this” is checked, then show the below question]
**What is the reason(s) that you have a problem?** [check all that apply]

- I don’t want my results to be reported to a public health agency.
- My doctor should have told me about this reporting before the HIV resistance test was done.
- Other: (specify)

1. **If the doctor had told you during your initial clinic visit that your HIV resistance test results would be sent automatically to public health agencies, how willing would you have been to take the test?**

- Very willing to have the resistance test done
- Somewhat willing to have the resistance test done
- Neither willing nor unwilling to have the resistance test done
- Somewhat unwilling to have the resistance test done
- Very unwilling to have the resistance test done

1. **You have just learned from a trusted media source—not the doctor—that HIV resistance test results are automatically reported to public health agencies. How likely are you to return to this doctor for continued care for HIV infection?**

- Very likely
- Somewhat likely
- Neither likely nor unlikely
- Somewhat unlikely
- Very unlikely

1. **In your opinion, how important is it for patients *to know* that their HIV resistance test results are reported automatically to public health agencies *before* they have the test done?**

- Extremely important
- Very important
- Moderately important
- Slightly important
- Not at all important
- ***of the other answers]***
