# Supplementary material for: Impact of Disclosing to Patients the Use of Antiretroviral Resistance Testing Results for Molecular HIV Surveillance: A Randomized Experiment in 2 National Surveys
Source: JMIR Public Health Surveill. 2025 Apr 11;11:e64663. doi: 10.2196/64663 (PMC12007841; doi:10.2196/64663)
Supplement: Multimedia Appendix 3 [file publichealth-v11-e64663-s003.docx]

**VIGNETTE SURVEY STUDY –COGNITIVE INTERVIEW QUESTIONS**

**VIGNETTE REVIEW**

*The interviewer will ask the participant to read the brief vignette after which the interviewer will assess the participant’s understanding of each scenario by asking a series of questions such as:*

- Could you tell me what this scenario is saying in your own words?
- Is there anything unclear or that you don’t understand? What could be made clearer?

**SURVEY ITEM ASSESSMENT**

*The interviewer will ask the participant to answer each of the survey items. After the participant completes the survey items, the interviewer will do a question by question inquiry, modeled on PREMIS Standard Cognitive Interview Probes *, to asses understanding, assumptions, response construction, and sensitivity.*

**Understanding**

- What is this question asking you?
- Can you put this question into your own words?
- When you heard this question, what were you thinking about when I said “X”? “X”=:
  - “HIV resistance test”
  - “HIV surveillance”
  - “automatically”
  - “genetic changes in the HIV virus”
- Additional probes:
  - “willing” – check if participants understand the new phrase (instead of comfortable)
  - “doctor” – do they confuse the doctor in this hypothetical scenario with their own actual primary care provider?
  - “trusted media source” – how do participants interpret this phrase?
- Disclosure Q2 – probe more and ask how participants understand the question and/or what the question is asking
- Is there a better way to ask this question?

**Assumptions**

- Does this question apply to you? (Probe whether participant understands the scenario is hypothetical and not asking about their personal doctor, care, etc.)

**Response Construction**

- How did you decide on that answer?
- What steps did you go through to get to that answer?
- Was it easy to pick one of the options?
  - [If no] What response options should the researchers use for this question?
- What were you thinking about when you selected “X” instead of “X”?

**Sensitivity**

- Is this question difficult to answer?
- Do any of the words in this question make you uncomfortable?
- Is it OK for researchers to ask patients a question like this on a survey?

**PREMIS (R21MH092253) Cognitive Interview Standard Probes, Version Date: April 4, 2011*
